# Effects of different treatments on pain, functional disability, position sense and range of motion in elite bodybuilders with chronic low back pain

**DOI:** 10.1038/s41598-024-59684-2

**Published:** 2024-04-22

**Authors:** Amirmohammad Faal Papoli, Seyed Mohammad Hosseini, Seyed Hossein Mirkarimpour

**Affiliations:** 1 Department of Physical Education and Sport Sciences, Faculty of Literature, Humanities and Social Sciences, Tehran Science and Research Branch, Islamic Azad University, Tehran, Iran; 2 Department of Sport Rehabilitation and Health, Faculty of Sport Sciences and Health, Shahid Beheshti University, Tehran, Iran; 3 Faculty of Physical Education and Sport Sciences, University of Tehran, Tehran, Iran

**Keywords:** Low back pain, Kinesio tape, Proprioception, Dry needling, Massage therapy, PNF, Health care, Medical research

## Abstract

Back pain is one of the major global challenges and is one of the most prevalent musculoskeletal disorders occurring in 80% of people at least once in their lifetime. Therefore, the need to find appropriate treatment methods for this issue is very important. The objective is to examine the short-term and acute effects of a treatment session with dry needling, massage therapy, stretching exercises and Kinesio tape on pain, functional disability, position sense and range of motion in elite bodybuilders with non-specific chronic low back pain. The sample of this quasi-experimental study consisted of 48 bodybuilders with non-specific chronic low back pain (all male, mean age = 25.96 ± 2.18 years; mean weight = 74.45 ± 4.51 kg; mean height = 173.88 ± 3.74 cm; mean BMI = 24.60 ± 0.74 kg/m^2^) who randomly were placed in 4 dry needling, massage therapy, stretching exercises and Kinesio tape groups. The duration of each intervention was 30 min. The dependent variables in this study included the massage range of motion, position sense tests and visual pain scale that were taken separately from each subject in pretest, posttest (acute effect) and follow-up test (72 h after posttest; short-term effect). The results of a 4 (groups) × 3 (time) the mixed ANOVAs showed that pain in the short-term phase was significantly lower in the dry needling group than in the stretching and massage groups (*P* < 0.05). Also in the acute effect phase, the flexion range of motion was significantly lower in the dry needling group than in the massage group (*P* < 0.05). Furthermore, the two groups of stretching and massage exercises showed significantly greater range of motion (*P* < 0.05). Other comparisons were not significant (*P* > 0.05). The findings of the study showed that both massage and stretching treatment have higher acute effects, while dry needling treatment was more effective in follow up. On the other hand, these findings show that these treatment methods can have immediate and lasting positive effects in improving the performance in elite bodybuilders with non-specific chronic low back pain.

## Introduction

Back pain is one of the major global challenges and is one of the most prevalent musculoskeletal disorders occurring in 80% of people at least once in their lifetime^1^. Although in most cases the back pain is resolved within 8 to 12 weeks, in some cases this pain becomes chronic and remains for more than 12 weeks^2^. This chronic and complex condition reduces the individual's efficiency and therefore leads to direct and indirect health care costs. 85% of chronic back pains do not have any specific diagnostic factor and are classified as non-specific chronic low back pain^3^. Considering the prevalence of low back pain in bodybuilders, studies have reported low back pain as one of the common problems in bodybuilders. For example, Pinto et al. reported a prevalence of 47.3% of low back pain in bodybuilders^4^. To this end, Silva et al. (2016) reported a prevalence of 38% and Santos et al. reported a prevalence of 57.1% of low back pain in individuals who did resistance exercise^5^. In a study conducted in 2017, the World Health Organization (WHO) reported that back pain-related disability increased by 17.5% over the ten years from 2007 to 2017^6^.

Low back pain can lead to many problems due to the pain experienced by people with back pain. People become less active because of the low back pain and this situation causes muscle imbalance^7^. That is, a group of muscles show increased activity and a group show decreased activity, which causes disturbance in the core muscle strength and endurance and the reduction of the range of motion in the spine, resulting in the problem in position sense^8,9^.

So far, many studies have investigated different treatment approaches for non-specific chronic low back pain^10–13^. Although there are many contradictions, such as the degree of effectiveness and their possible side effects, regarding the selection of the most favorable treatment approach, there is an agreement that such advices as bed rest, which were previously recommended for acute back pain, are not very effective for this type of back pain^10,11^. Based on the research findings, various treatment approaches have been used to treat back pain, including drug therapy^12^, dry needling^13,14^, kinesiotaping^15,16^, laser therapy^17,18^, massage, hydrotherapy^19,20^, ultrasound^21,22^, and finally surgery^23–25^. Based on these studies [], it has been determined that each of these treatment methods can have immediate or delayed effects on the recovery of people with low back pain.

In recent years, the number of studies investigating non-surgical treatments in the treatment of patients with low back pain has increased, which implies the importance of this fact^10,26,27^. One of the treatments for low back pain that has received attention recently is massage therapy^28^. Previous studies have reported that the mechanism of pain reduction after massage is caused by the endogenous system, blocking impulses to the brain and the release of endorphins^28^. For example; Furlan et al. (2015), in a review article and with a review of 25 articles showed that massage therapy, as an efficient and available method, can be one of the prominent methods in improving performance and reducing pain in people with chronic low back pain^19^. Kinesio tape has also been used to improve back pain, and its benefits include improving function, increasing blood flow, reducing pain, correcting joint malalignment, and lifting the skin and creating more space under the taped area^29^. For example; Mohammad et al. (2023) by examining the effectiveness of the Kinesio taping method on improving performance and reducing pain in 60 male participants, they showed that the Kinesio taping method can reduce pain, improve performance and range of motion of people after 2 to 6 weeks of training^16^.

It has also been reported in past research that many people with back pain may have decreased range of motion in the hamstring muscles, which can be said to be likely to reduce back pain by stretching these muscles^30^. Dry needling treatment is also a treatment method that is used to treat a variety of pain syndromes, and there is a growing body of articles that examines its clinical effectiveness in the treatment of musculoskeletal pain^31^. Dry needling causes a change in the blood flow of the muscles, and with this approach, it can have a soothing effect on pain^31^. For example, Rajfur et al. (2022) showed that dry needling interventions given as a supplement to a regular exercise program can lead to pain reduction in people with chronic low back pain^13^.

Although many studies have so far investigated the effectiveness of these mentioned treatment methods on improving performance and reducing pain in people with chronic low back pain^12–23^, but to the best of our knowledge, there is no research on the simultaneous comparison of these methods and its effects on people with chronic low back pain. Also, this research is one of the first studies that examines the immediate and lasting effects of these methods in elite athletes. Therefore, it seems that conducting the present study is of great importance and its results can be helpful for the community of body builders who mainly face pain caused by chronic low back pain. According to the results of previous similar researches^12–23^, in this research it is assumed that all treatment methods can have significant effects on improving motor function and reducing pain in elite bodybuilders with non-specific chronic low back pain. Consequently, the present study aims to compare the acute and short-term effect of a treatment session with dry needling, massage therapy, stretching exercises, and Kinesio tape on pain, function disability, position sense, and range of motion in elite bodybuilders with non-specific chronic low back pain.

## Materials and methods

### Research design

In this quasi-experimental study, the research samples and the data analyzer were blinded. Firstly, written consent form was given to the samples and the samples were included in the research process with their informed consent. The study design was approved by the ethics committee of Islamic Azad University Science and Research Branch (Approval Code: (IR.IAU.SRB.REC.1401.096; Approval Date: 27.11.2022) and was implemented according to the Declaration of Helsinki.

### Participants

The statistical population of the present study included male bodybuilders with an age range of 20–30 years (all male, mean age = 25.96 ± 2.18 years; mean weight = 74.45 ± 4.51 kg; mean height = 173.88 ± 3.74 cm; mean BMI = 24.60 ± 0.74 kg/m^2^) who suffered from non-specific chronic low back pain. These participants were examined by an orthopedic doctor and were identified as people with non-specific chronic low back pain based on these examinations. Participants were 48 male bodybuilders with non-specific chronic low back pain who met the research inclusion criteria were included in the research process as the research sample and were randomly divided into four groups of 12 people treated with dry needling, massage, stretching exercises, and Kinesio tape. It should be noted that the sample size was considered as 40 based on GPower software (effect size: 0.32, alpha error 0.05 and 95% power) with the possibility of dropping out, a total of 48 people in 4 groups of 12 people were considered as the sample^14^. Research inclusion criteria include: Being between 20 and 30 years old, being male, having back pain for a period of at least 12 weeks, having back pain in the range between 3 and 7 in the visual pain scale, continuous engagement in the field of bodybuilding, not having symptomatic disc herniation with pain caused by Nerve root involvement, not suffering from spondylosis and spondylolisthesis, not having a history of spine surgery, not performing a specific treatment protocol or physiotherapy in the last 3 months, not having any abnormalities or damage affecting the research process and not having structural osteoporosis of the spine. However, due to the fact that the current research was quasi-experimental, it was not possible to control all the co-variables. Therefore, during the experiment, it is assumed that all participants had normal sleep, nutrition and physical activity. The research exclusion criteria included: the individual's unwillingness to continue participation, failure to participate in the sessions of measurement of the research dependent variables in the pre-test phase, the acute effect investigation or durability and skin sensitivity to the Kinesio tape.

### Procedure

In the first step, male bodybuilders suffering from non-specific chronic low back pain were sampled using purposive convenience sampling method, and if they agreed to cooperate, they were approved by a specialist doctor. In the next step, the selected individuals were divided into four exercise groups, including dry needling, massage, stretching exercises, and Kinesio tape based on the degree of pain and demographic information. The duration of each intervention was 30 min for all methods. A description of the research process was explained for all the subjects. Then, range of motion and position sense tests were taken separately from each subject, and the visual pain scale was completed by the subjects and the results were saved as pre-test data. Afterward, each subject received the treatment corresponding to his assigned group. Immediately after completing the treatment, all the tests were repeated and the results of this stage were saved as the acute effect data. After this stage, the subjects continued their normal life, and after 72 h, all the tests were repeated and the short-term effect data. The research included pretest (first day), intervention (first day; 30 min for all methods), acute posttest (first day) and short-term posttest (after 72 h).

#### Massage protocol

In this research, The Massage protocol was administered to the participants on the dorsal region of the trunk extending from occiput to axillary lines and until posterior iliac crest.

**Figure 1:**
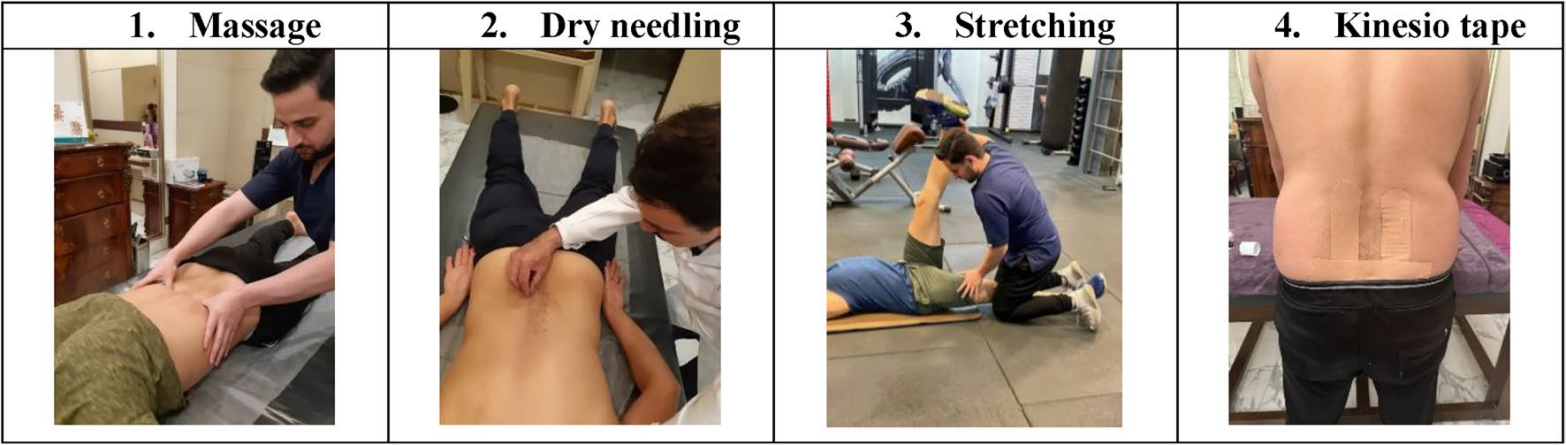
The image of the performed interventions. **1** The massage intervention, **2** The dry needling intervention, **3** The stretching intervention, **4** The Kinesio tape intervention.

Effleurage, Compression and Static Contact, Petrissage, Kneading and finally Friction were used (Table 1; Fig. 1)^32^.

**Table 1:** Description of the Massage Therapy Technique Applied to Study Participants.

Position of the participant	All the participants were positioned in prone position on a manual therapy bed with pillows supported underneath the lower legs				
Massage Techniques	Effleurage: Cycles of pressured long gliding stokes with drainage towards the axillary lymph nodes	Compression & Static Contact: Moderate compressive pressure applied through the palm and heel of the hand in a slow sustained pumping method	Petrissage: Pick up and squeeze techniques with mild to moderate pressure	Kneading: Moderate pressured deep circular movements performed through fingers, palm and heel of the hand	Friction: Quicker deeper movements performed on the tissue perpendicular to the direction of the muscle fibres
Duration of Application	6 min, 3 min duration × 2 cycles at the beginning and end of the session	3 min per cycle of intervention	5 min per cycle of intervention	5 min per cycle of intervention	1 min per cycle of intervention

#### Dry needling

A total of 15 needles were inserted. Five needles were inserted to the left and next to the pedicles (with a distance of about 25 mm) from L1 to L5 respectively, and five needles were inserted in the same place on the right side. In addition, five needles were placed between the spinous processes of the vertebrae from T12-L1 to L4-L5. The session lasted for 8 min. A stopwatch was used to estimate the time^33^ (Fig. 1).

#### Stretching exercises

Stretching exercises included PNF stretching movements on the hamstring muscles. In this group, a 5-min warm-up, including soft jogging, was performed at the beginning. The stretching program consisted of three rounds of stretching with a 15-s rest period between each stretch. Each stretching round consisted of three stretching repetitions. The subject was in the supine position and the pelvic was fixed on the anterior superior iliac spine. The subject's leg was raised completely straight from the knee (in the full extended) by the researcher to the area where movement is limited. Then, with the help of the researcher, an isometric contraction of the hamstring muscles was taken for 7 s. The subject's leg remains in the same position for a second in a relaxed state and was taken to the new movement limit point and subjected to stretching for 7 s, and then isometric contraction was taken. This process was repeated for 3 stretches and 3 contractions in each leg. This movement was repeated for both legs^34^ (Fig. 1).

#### Kinesio tape protocol

According to Kenzo Kase's Kinesio Taping, patients were taped^35^. An expert physical therapist taped patients. In a standing position, kinesio taping was put on the sacrum and both sides of the paravertebral muscles. We used four I-shaped Kinesio tapes that were 5 cm wide and 0.5 mm thick. As the lumbar flexion reached its peak and rotated to the opposite side, two bands were applied. These bands were placed on each paravertebral muscle with 10% to 15% tension, vertically from the lower posterior iliac crest region to the upper twelfth rib region (paper-off tension). The remaining 2 bands were attached diagonally on the sacrum, with 50% to 75% of tension^35,36^ (Fig. 1).

#### Pain assessment

The visual pain scale is a 10 cm horizontal line, at the left end of which is the word “no pain” and at the right end of it is the word “pain as bad as it could possibly be”, and between these two, the pain intensity increases from 1 to 9. Pain scale was completed using this visual scale by the subject in two stages before and after the intervention^37^.

#### Evaluation of spine’s range of motion

The flexion and extension range of motion of the lumbar spine was evaluated using the modified Schober test. With the patient in standing position, the middle point of the superior posterior articular spines is marked as the first point and 15 cm above is marked as the second point. By fixing the pelvis, the subject is asked to perform the most flexion and extension movements and the changes that occurred are recorded^38^.

#### Evaluation of proprioception

In this study, the ability of active reconstruction at an angle of 30 degrees was evaluated using a hand-held goniometer according to the method proposed by Newcomer et al. The reliability of this method is reported as 87%. First, markers were attached to the middle of the upper outer surface of the humerus, the ridge of the iliac crest, and the upper outer surface of the hip joint. The subjects were placed in a comfortable standing position on both legs without shoes and socks. The legs were shoulder width apart. The hands were placed in a crossed position in front of the shoulder (so as not to use the contact of the palm with the front surface of the thigh during bending as a guide to reach the target angle), the neck was kept in a natural position, and the eyes were closed with a blindfold to remove visual afferents. Then, the center of the goniometer was placed on the iliac crest, and the two arms of the goniometer was set on the marker on the outer part of the thigh, and the other arm was set to 30 degrees of bending, and the subject was asked to bend to 30 degrees with his eyes closed and at a uniform and relatively slow speed and try to remember this position with a 5-second pause (at this stage, the subject was informed of the end of the movement by sound stimulation), then he slowly returned to the initial position and after pausing for 5 s, he started the next movement. After three repetitions (for learning) in the test phase, the person had to recreate the 30-degree bent position (without sound stimulation). This test was repeated three times, then the average amount of error in reconstructing the situation in three repetitions was recorded as the amount of reconstruction error. Then, the average value of the absolute error in the reconstruction of the position in three repetitions was recorded as the position reconstruction error rate^39^.

### Statistical data analysis

The information obtained and recorded from the evaluations was analyzed using SPSS version 26 software. The normality of data distribution was checked by the Shapiro–Wilk test and the homogeneity of variance was checked by the levene's test. To investigate the between and within group changes, and the interaction effect of time and group, the 4 (groups) X 3 (time) the mixed ANOVA test was used. Then, in order to more accurately investigate the between group difference, first, one-way ANOVA test was used, and then Tukey's post hoc test was applied. Paired sample t-test was used to examine within group changes. The significance level in this research was considered as 0.95 and alpha was considered smaller than 0.05.

### Ethical considerations

In order to conduct the study, approval was obtained from the Research Ethics Committee of Islamic Azad University Science and Research Branch (IR.IAU.SRB.REC.1401.096), and the institution where the study was conducted. The patients included in the study were informed about the aim of the study and their questions were answered. The patients were informed that their information would be kept confidential and would not be used elsewhere and that they had the right to withdraw from the study whenever they wanted. The patients filled out a voluntary consent form declaring that they accepted to participate in the study voluntarily. Since the inclusion of the human phenomenon in the study requires the protection of individual rights, the relevant ethical principles of “Informed Consent”, “Voluntary Basis” and “Protection of Confidentiality” were adhered to.

###  Ethical approval

The study design was developed based on the Declaration of Helsinki and approved by Islamic Azad University Science and Research Branch’s Ethics Committee (Approval Code: (IR.IAU.SRB.REC.1401.096; Approval Date:27.11.2022). Informed consent was obtained from all subjects involved in the study.

### Informed consent

Informed consent was obtained from all participants to publish their identifying information/images.

## Results

The information related to the age, height, weight, and body mass index of the subjects is presented as four separate groups in Table 2, and the comparison of the four groups through the one-way analysis of variance test showed the homogeneity of the four groups in terms of demographic characteristics. Also, before the start of the interventions, the research groups were compared in terms of pain, range of motion, flexion and extension, and sense of position, and the results of the one-way analysis of variance test showed that there was no significant difference between the research groups before the intervention (*p* > 0.05).

**Table 2:** Initial demographic characteristics of the study groups.

Variable	DN group	Taping group	PNF group	Massage group	*p*
	Mean ± SD	Mean ± SD	Mean ± SD	Mean ± SD	
Age	25.90 ± 2.42	26.63 ± 1.68	25.50 ± 2.22	25.75 ± 2.45	0.67
Weight	173.81 ± 3.78	173.54 ± 4.29	174.00 ± 4.05	174.16 ± 3.37	0.98
Height	73.45 ± 4.74	74.27 ± 4.92	75.00 ± 4.96	75.08 ± 3.89	0.82
BMI	24.29 ± 0.89	24.63 ± 0.80	24.74 ± 0.75	24.73 ± 0.48	0.45

*P* < 0.05.

The normality of the data was checked through the Shapiro–Wilk test, and the results showed that all variables have a normal distribution. The homogeneity of variance was also checked through the Levene’s test, and the results of this test indicated that the condition of variance homogeneity was met in all variables.

The results of repeated measures ANOVA test indicated a significant difference in the pain scale variable within the group [F(2,80) = 132.926, *p* < 0.05]. Also, there is a significant interaction between the group and research phases in terms of the pain scale variable [F(2,80) = 2.955, *p* < 0.05]. However, the effect of between-group factor related to pain scale variable is not significant [F(3,40) = 0.526, *p* > 0.05]. In the flexion range of motion variable, the results showed that there is a significant within group difference [F(2,80) = 179.832, *p* < 0.05] and there is a significant interaction between the group and research phases in terms of the flexion range of motion variable [F( 2,80) = 22.762, *p* < 0.05]. However, the effect of the between-group factor related to flexion range of motion variable is not significant [F(3,40) = 1.275, *p* < 0.05]. There was a significant within group difference in terms of the extension range of motion variable [F(2,80) = 309.025, *p* < 0.05] and there is a significant interaction between the group and research phases in terms of the extension range of motion [F(2,80) ) = 55.602, *p* < 0.05], while the effect of the between-group factor related to the extension range of motion variable is not significant [F(3,40) = 1.190, *p* > 0.05]. Also, the results showed that there is a significant difference in the position sense variable within the groups [F(2,80) = 132.926, *p* < 0.05] and there is a significant interaction between the group and research phases in terms of the position sense variable [F(2), 80) = 2.955, *p* < 0.05]. Finally, it was observed that the between-group effect related to the variable of position sense is not significant [F(3,40) = 0.526, *p* > 0.05].

According to the results of repeated measures ANOVA test, in order to conduction within-group comparison between different phases of the research, Bonferroni's post hoc test was used, the results of which are presented in Table 3.

**Table 3:** Results of Bonferroni's post hoc test.

variable	Group	Before intervention	Immediately after intervention	P1	72 h after intervention	P2	P3
		Mean ± SD	Mean ± SD		Mean ± SD		
VAS	DN group	5.45 ± 1.51	4.27 ± 1.10	0.001*	3.00 ± 1.34	0.001*	0.001*
	Taping group	5.63 ± 1.43	5.18 ± 1.25	0.046*	3.81 ± 0.87	0.001*	0.001*
	PNF group	5.40 ± 1.57	4.30 ± 1.49	0.001*	4.40 ± 1.26	0.001*	1.000
	Massage group	5.41 ± 1.37	3.88 ± 1.19	0.001*	4.33 ± 0.98	0.001*	0.020*
Flextion ROM	DN group	1.81 ± 0.33	2.01 ± 0.31	0.001*	2.24 ± 0.32	0.001*	0.001*
	Taping group	1.83 ± 0.36	1.89 ± 0.33	0.883	2.24 ± 0.24	0.001*	0.001*
	PNF group	1.87 ± 0.29	2.40 ± 0.34	0.001*	2.27 ± 0.33	0.001*	0.001*
	Massage group	1.88 ± 0.28	2.45 ± 0.35	0.001*	2.24 ± 0.34	0.001*	0.001*
Extention ROM	DN group	0.82 ± 0.16	0.86 ± 0.18	0.132	1.02 ± 0.18	0.001*	0.001*
	Taping group	0.80 ± 0.19	0.84 ± 0.18	0.132	1.03 ± 0.18	0.001*	0.001*
	PNF group	0.84 ± 0.15	1.11 ± 0.16	0.001*	1.02 ± 0.16	0.001*	0.001*
	Massage group	0.81 ± 0.15	1.14 ± 0.16	0.001*	1.02 ± 0.16	0.001*	0.001*
JPS	DN group	5.12 ± 1.07	4.45 ± 1.05	0.001*	3.84 ± 0.98	0.001*	0.001*
	Taping group	5.18 ± 0.84	4.99 ± 0.99	0.149	4.39 ± 0.72	0.001*	0.001*
	PNF group	5.39 ± 0.76	5.10 ± 0.67	0.009*	4.90 ± 0.76	0.001*	0.067
	Massage group	5.05 ± 0.81	4.83 ± 0.77	0.042*	4.55 ± 0.93	0.001*	0.002*

P^1^ = Comparison of Before intervention and Immediately after intervention. P^2^ = Comparison of Before intervention and 72 h after intervention. . P^3^ = Comparison of Immediately after intervention and 72 h after intervention.

*p < 0.05 is significant.

The results of the Bonferroni's post hoc test showed that the two dry needling and taping groups had significantly less pain in the acute phase and 72 h after the intervention compared to the pre-test, and also significantly less pain in the 72 h after the intervention compared to the acute phase. However, in the stretching exercises group, significantly less pain was reported in the acute phase than before the test and 72 h after the intervention, and pain was reported significantly less in the 72 h after the intervention than before the test. In the massage group, the pain was less than the pre-test in both the acute and 72 h after the intervention, while no significant difference was observed between the acute phase and 72 h after the intervention.

In the two groups of dry needling and taping, higher range of motion in flexion and extension movements and less error in position sense were observed in 72 h after the intervention compared to the acute and pre-test phases. However, the massage and stretching exercise groups showed higher range of motion in the acute phase and less error in the position sense than the pre-test phase and 72 h after the intervention.

The results of the one-way analysis of variance test also showed that there was a significant difference between the research groups only in the pain variable at 72 h after the intervention and in the range of motion variable at the acute phase (Table 4). The follow-up test results showed that regarding the pain variable, the dry needling group had significantly less pain 72 h after the intervention compared to the two massage and stretching exercise groups. However, in the range of motion variable, a significantly greater range of motion was observed in the extension movement of the massage and stretching exercise groups than the taping and dry needling groups, and in the flexion range of motion, it showed a greater range than the taping group (Table 5).

**Table 4:** Results of the one-way analysis of variance test.

Variable	Group	Sum of squars	DF	Mean of squars	f	*p*	µ
VAS	Before intervention	0.388	3	0.129	0.060	0.981	0.004
	Immediately after intervention	10.847	3	3.616	2.275	0.095	0.146
	72 h after intervention	13.729	3	4.576	3.610	0.021*	0.213
Flextion ROM	Before intervention	0.038	3	0.013	0.123	0.946	0.009
	Immediately after intervention	2.613	3	0.871	7.648	0.001*	0.366
	72 h after intervention	0.007	3	0.002	0.024	0.995	0.002
Extention ROM	Before intervention	0.006	3	0.002	0.072	0.975	0.005
	Immediately after intervention	0.825	3	0.275	9.732	0.001*	0.05
	72 h after intervention	0.001	3	0.0001	0.019	0.996	0.422
JPS	Before intervention	0.697	3	0.232	0.296	0.828	0.022
	Immediately after intervention	2.589	3	0.863	1.135	0.347	0.07
	72 h after intervention	6.115	3	2.038	2.727	0.057	0.174

* = *P* < 0.05.

**Table 5:** Results of the Tukey post hoc test.

Variable	Group	Mean difference	*P*
VAS 72 h after intervention	DN VS Taping	− 0.818	0.577
	DN VS PNF	− 1.400	0.042*
	DN VS Massage	− 1.333	0.043*
	Taping VS PNF	− 0.582	1.000
	Taping VS Massage	− 0.515	1.000
	PNF VS Massage	0.067	1.000
Flextion ROM Immediately after intervention	DN VS Taping	0.127	1.000
	DN VS PNF	− 0.382	0.079
	DN VS Massage	− 0.440	0.019*
	Taping VS PNF	− 0.509	0.008*
	Taping VS Massage	− 0.567	0.001*
	PNF VS Massage	0.058	1.000
Extention ROM Immediately after intervention	DN VS Taping	0.018	1.000
	DN VS PNF	− 0.246	0.011*
	DN VS Massage	− 0.278	0.002*
	Taping VS PNF	− 0.265	0.005*
	Taping VS Massage	− 0.396	0.001*
	PNF VS Massage	0.032	1.000

* = *P* < 0.05.

## Discussion

The results of the study showed a significant reduction in the pain, range of motion and position sense in elite bodybuilders with non-specific chronic low back pain immediately after taping by kinesiotape, which is consistent with the findings by Castro et al. (2012)^29^, and Lee et al. (2012). The findings by Parreira et al. (2014)^30^ and González Enciso (2009)^31^ were not consistent with the present study, which could be due to the small sample size (4 people) and shorter duration in our study. Neurophysiological studies have associated pain in the lumbar spine region with disruption of mechanoreceptor and possibly with upper extremity proprioception impairment. Kase et al. (1996) presented a theoretical framework that explained the reduction in pain-related disability in the spine immediately after taping^32^. They reasoned that when the muscle contracts, receptors in the Golgi body are stimulated and transmit information to the central nervous system, where motor inhibitory neurons are active. Also, taping by stimulating the receptors of the Golgi body causes the beginning of this process. Another possible mechanism is that the taping pulls the upper layers of the skin and creates more space between the middle skin membrane and the muscle.

This created space reduces the pressure on the lymph channels in the area between the muscle and the middle membrane of the skin, creating more space for lymph flow and thus the lymph can flow better in an injured area. This space also contains various nerve receptors that send specific information to the brain. Taping modulates the information that these receptors send to the brain and causes less reactive responses in the body. According to previous studies, the short-term improvement in pain level after kinesiotape protocols in patients with chronic back pain can be caused by the gate or valve control theory of pain. According to this theory, any contact stimulation activates neural mechanisms in the posterior horn of the spinal cord, which can act as a gate or valve and modulate the nerve flow from the peripheral fibers to the center. Therefore, kinesiotape can reduce pain in patients with non-specific chronic back pain by stimulating the contact it creates on the skin and muscles through the mechanism of releasing hormones of the pain suppression system, such as endorphin and enkephalin^33^. On the other hand, kinesiotape can increase stability in joints and strengthen weak muscles in patients, all of which can lead to a reduction in pain after the intervention^34^. In addition, some of the positive effects of kinesiotape may indirectly affect pain and disability improvement, such as normalizing muscle tone, improving postural control, range of motion, circulation, and proprioception. Reducing pain can lead to improvement of range of motion, because pain is an important factor in reducing range of motion; therefore, by reducing the amount of pain, as well as improving function disability and reducing muscle spasm, an improvement in range of motion is also observed. Taping increases proprioception by tensioning the input tip and improves perception of body movement even after the tape is removed.

The potential mechanism by which kinesiotape improves proprioception is not yet known. Some authors have hypothesized that the cutaneous feedback provided by the kinesiotape can be enhanced. The pressure and tension exerted due to the application of kinesiotape on the skin during intense movement, similar to the joint mechanoreceptors, can also stimulate the skin mechanoreceptors and provide signal information of joint movement or joint position^35^.

Regarding the effect of dry needling, the results of the study showed a significant reduction in the amount of pain, range of motion and position sense in elite bodybuilders with non-specific chronic low back pain after dry needling, which is consistent with the findings Tedoros et al. (2020) ^44^, Hong Teng et al. (2018)^29^.

Regarding the effect of dry needling on pain, it has been suggested that it may be related to the release of myofascial trigger points (TrPs) and thus improving the lateral bending ability. This method is generally performed in muscle areas with myofascial trigger points in an effort to reduce muscle tension, restore muscle function, and relieve pain. It makes sense that muscles that work to control postural adjustments, contraction or relaxation, are better tuned at lower levels of perceived pain. Hence, at lower levels of pain, agonist and antagonist muscles may be better coordinated, at least as can be determined from the results of the dynamic balance task. It is expected that the “release” of the lumbar muscles has a significant effect on the sensorimotor system. However, dry needling may be beneficial to the muscles, giving a “window” to the motor system to test, train, and develop new movement patterns. Also, the large effect size in pain reduction one week after dry needling shows that pain reduction continues over time. It can be concluded that a significant further reduction in pain intensity can be expected beyond the acute effects of dry needling at least one week later in patients with chronic low back pain. It is possible that mobility limitation due to pain rather than muscle dysfunction may account for greater improvements in range of motion after dry needling intervention. Loss of muscle spindle sensitivity and muscle response can occur following muscle damage, which can cause proprioceptive signaling disorders and motor control disorders. Using dry needling, through the normalization of muscle spindle activity to optimize sensory information to the central nervous system, leads to increasing the accuracy of the information ascending to supraspinal structures and improving descending output. Dry needling balance theory shows that dry needling interventions in peripheral muscles can lead to peripheral and central action mechanisms on motor efficiency and physical performance^45^.

Regarding the effectiveness of massage, the results of the study showed a significant reduction in the amount of pain, range of motion, and position sense in elite bodybuilders suffering from non-specific chronic low back pain after massage, which is similar to the findings by Forlan et al. (2008)^46^, Cherkin et al. (2009)^47^, and Nazemzadeh et al. (2012) ^48^. The reason for the reduction of pain after massage can be due to the reduction of activity of nerves, reduction of muscle excitability, stimulation of alpha fibers and regulation of muscle tone^49^. Also, due to the fact that one of the common causes of pain in people with back pain is the presence of trigger points, and considering that trigger points send many impulses to the nervous system and cause a cycle of spasm and pain spasm, placing the muscles in a short length causes the impulse sending from the trigger points to stop and can cause it to relax and ultimately relax the entire muscle^50^. Massage creates a positive emotional state in the area with regular and rhythmic movements that can relieve the patient's stress and anxiety caused by pain. Following this, spasms and muscle cramps are partially resolved and the tissue relaxes under the masseuse's hands, breaking the vicious cycle of pain-spasm. According to the stated theory, massage improves blood circulation and lymph flow, brings nutrients and fresh oxygen to the tissues, and helps to remove toxic substances. Massage, by increasing blood circulation, increasing the pain threshold in receptor stimuli and increasing the release of hormones such as norepinephrine, cortisol, endorphin, and serotonin, reducing stress, fatigue, depression and chronic pain levels and increasing the sense of health and well-being in the body of patients, plays an important role to solve these obstacles and improve neural connections^51^.

Also, regarding the effectiveness of stretching, the results of the study showed a significant reduction in the amount of pain, range of motion, and position sense in elite bodybuilders with non-specific chronic low back pain after stretching, which is consistent with the findings by Bahaduria and Gorodut (2017) 52], and Shamsi et al. (2020)^53^. It was observed that immediately after passive stretching, muscle stiffness decreased as the range of motion increased. In this case, the stiffness changes increase the effectiveness of passive stretching and further increase the range of motion. According to Shalmars (2004), the muscle spindles and the Golgi organ have a close cooperation so that the first one causes muscle tension to produce smooth and rhythmic movement, and the second one causes the muscle to rest and does not allow inappropriate pressure and excessive stimulation to the muscle^54^. Therefore, with the step-by-step implementation of the massage method, patients are able to benefit from this method and are treated, without having a negative reflection in the gradual course of the range of motion. The effect of stretching exercises on proprioceptive neuromuscular facilitation in increasing flexibility involves neurophysiological mechanisms, one of which is the muscle stretch reflex. These mechanisms increase the tolerance of tension, which is achieved during the increase of muscle strength or the reduction of pain.

Research has shown that performing exercises that improve neuromuscular control, including proprioceptive neuromuscular facilitation exercises, accelerates the achievement of a relative degree of flexibility compared to other stretching exercises and is more suitable for treating and rehabilitating pelvic floor muscle injuries^55^. In this way, by performing this method, the hamstring muscle fibers are stretched and due to the changes in its central part, an impulse is created, and these impulses are sent to the central nervous system. Proprioceptive neuromuscular facilitation (PNF) exercises are designed to enhance the response of neuromuscular mechanisms by stimulating proprioceptive receptors. Patterns of PNF exercises have a spiral and diagonal direction, and the performance of these patterns is in accordance with the topographic arrangement of the muscles used. Performing movements in PNF patterns may allow muscles to function in ways that closely approximate actions and movements found in various sports^56^. The results of this study cannot be generalized to all of the people with low back pain because the sample in this study was elite bodybuilders with non-specific chronic low back pain.

## Conclusion

The results showed that the amount of pain in the short-term phase was significantly lower in the dry needling group than in the stretching and massage groups (*P* < 0.05). Also, the results of Tukey's post hoc test showed that, in the acute effect phase, the flexion range of motion was significantly lower in the dry needling group than in the massage group. Furthermore, the two groups of stretching and massage exercises showed significantly greater flexion range of motion than the tapping group at this stage (*P* < 0.05). However, there was no significant difference between the other groups in this phase (*P* > 0.05). Also, in the acute effect phase, the extension range of motion was significantly higher in the two groups of stretching exercises and massage than in the two groups of taping and dry needling (*P* < 0.05). However, there was no significant difference between the research groups in the position sense and function disability variables. The results showed that massage and stretching movements have higher acute effects, while dry needling was more effective in follow up.

## Data Availability

The datasets used and/or analysed during the current study available from the corresponding author on reasonable request.
